# Investigation of the response of *Platycodongrandiflorus* (Jacq.) A. DC to salt stress using combined transcriptomics and metabolomics

**DOI:** 10.1186/s12870-023-04536-w

**Published:** 2023-11-25

**Authors:** Meixi Zhang, Yushu Xing, Jiannan Ma, Ying Zhang, Juan Yu, Xiaoqin Wang, Xin Jia

**Affiliations:** College of Pharmacy, Inner Mongolia Medical University, Hohhot, China

**Keywords:** *Platycodon grandiflorus*, Salt stress, Transcriptomics, Metabolomics, Platycodin D

## Abstract

**Background:**

*Platycodon grandiflorus* (Jacq.) A. DC is a famous traditional Chinese medicine in China and an authentic medicine in Inner Mongolia. It has been traditionally used as an expectorant in cough and also has anti-inflammatory and other pharmacological effects. As a homologous plant of medicine and food, *P. grandiflorus* is widely planted in Northeast China. Soil salinity isa limiting factor for its cultivation. In this study, we comprehensively described the physiological characteristics of *P. grandiflorus* and combined transcriptomics and metabolomics to study the response of roots of *P. grandiflorus* to salt stress.

**Results:**

Overall, 8,988 differentially expressed genes were activated and significantly altered the metabolic processes. In total, 428 differentially abundant metabolites were affected by salt stress. After moderate and severe salt stress, most of the differentially abundant metabolites were enriched in the L-phenylalanine metabolic pathway. Through the comprehensive analysis of the interaction between key genes and metabolites, the main pathways such as lignin compound biosynthesis and triterpene saponin biosynthesis were completed. The relative content of compounds related to lignin biosynthesis, such as caffeic acid, coniferin, and syringing, increased under salt stress, and the related genes such as *PAL*, *C4H*, and the key enzyme gene *UGT72E2* were activated to adapt to the salt stress. Platycodon saponin is one of the major triterpene saponins in *P. grandiflorus*, and Platycodin D is its most abundant major bioactive component. Under severe salt stress, Platycodin D level increased by nearly 1.77-fold compared with the control group. Most of the genes involved insynthetic pathway of Platycodin D, such as *HMGCR*, *GGPS*, *SE*, and *LUP*, were upregulated under salt stress.

**Conclusion:**

Salt stress led to a decrease in the biomass and affected the activities of antioxidant enzymes and contents of osmotic regulators in the plant. These results provided not only novel insights into the underlying mechanisms of response of *P. grandiflorus* to salt stress but also a foundation for future studies on the function of genes related to salt tolerance in the triterpenoid saponin biosynthesis pathway.

**Supplementary Information:**

The online version contains supplementary material available at 10.1186/s12870-023-04536-w.

## Background

Soil salinization is the process in which the salt content of soil surface layer continuously increases to exceed a certain limit under natural and anthropogenic effects [1]. Soil salinization can lead to the decline in crop yield and limit planting capacity. It is a serious problem worldwide and one of the important abiotic stresses faced by plants. Salt tolerance in plants is a very complex phenomenon involving many biological processes and pathways operating at the whole plant level [2]. Therefore, the response of plants to salt stress and mechanism of salt tolerance in plants should be studied to discover the plant genes related to salt tolerance and to further develop and utilize salt-tolerant resources in plants to enhance salt tolerance of plants for comprehensive utilization of saline–alkali soils.

Salt stress can inhibit crop growth and eventually lead to crop yield reduction [3]. The underlying mechanisms are mainly attributed to two aspects: osmotic pressure and ionic toxicity [4]. Osmotic stress is salt-stress-induced reduction in plants’ ability to absorb water, leading to the inhibition of plant growth. Osmotic stress is the first type of stress that occurs when plants are exposed to saline soil, and it directly affects plant growth. High concentration of salt ions decreases the osmotic potential of external soil; when it exceeds a certain threshold, plants cannot maintain ion stability and growth, resulting in ion toxicity. In severe cases, it may inhibit cell division and growth, lead to the separation and death of cell plastids, inhibit seed germination, reduce photosynthetic rate, and affect plant growth. In addition, high salinity can cause secondary stress, leading to the accumulation of reactive oxygen species (ROS) [5] in plants. This results in oxidative stress and damage to cellular proteins, nucleic acids, and other biomacromolecules, causing irreversible damage.

Salt stress produces excessive ROS, whose strong oxidative capacity leads to peroxidation of unsaturated fat in cell membranes, destruction of membrane structure, and production of malondialdehyde (MDA). The content of MDA reflects the degree of damage to membrane structure. ROS can activate certain enzymatic and nonenzymatic systems, which act to reduce oxidative stress [6]. The enzymatic system is mainly composed of superoxide dismutase (SOD), peroxidase (POD), and catalase (CAT). These enzymes scavenge the ROS. Nonenzymatic system consists of metabolites, such as ascorbic acid and lignin, formed because of salt stress. Enzymatic and nonenzymatic systems play an important role in scavenging ROS [5] and reducing the damage due to ROS to plant cells. In addition, the content of osmoregulatory substances is affected by salt stress. Proline has a certain osmotic protection to salt stress. Proline protects cell membrane by reducing cell osmotic potential and makes antioxidant enzymes more stable. Soluble sugar is a substance synthesized by plants under stress, which maintains the osmotic balance of cells and reduces salt stress to a certain extent.

Various omics techniques have been developed to study the complex biological mechanisms of various plants. They have been proved to be beneficial to analyze the changes in DNA, transcripts, proteins, and metabolites in the context of environmental and physiological stresses. The omics techniques are convenient for exploring the molecular mechanism of plant development and heredity [7]. Among them, genomics, transcriptomics, proteomics, and metabonomics are the current research hotspots [8–10]. These techniques interpret the growth and development of organisms at different levels. However, it is difficult to describe the complex process of plant biology using a single omics technique. The multiomics method can identify the candidate key factors by integrating the information obtained from various omics methods. By integrating information at the gene, transcription, protein, and metabolism levels and building a gene regulation network, the regulation and causality of various molecules can be studied in detail to further better understand the gene function and interaction network of plants under different physiological and environmental stresses [9]. In recent years, the rapid development of high-throughput technologies has made the acquisition of transcriptome and metabolome data more rapid and economical and has deepened the understanding of various molecular regulatory mechanisms and metabolite regulatory networks in plants. In addition, multi-omicshas been applied to study plant stress resistance. Transcriptome and metabolome analyses revealed that salt and alkali stresses induced differentially expressed genes (DEGs) that participated in various biological processes of grapes and had different molecular functions [11]. Transcriptome and metabolome analyses revealed that under salt–alkali stress, the stress pathway of poplar was not through overexpression but through osmotic adjustment, ion compartmentalization, and ROS removal. The results revealed that the adaptation of poplar to salt–alkali environment was closely related to the high energy consumption and loss of transcriptional regulation [12]. In the combined transcriptome and metabolome analyses, association analysis between DEGs in the transcriptome and differentially abundant metabolites detected by the metabolome analysis allowed analysis of intrinsic changes in plants at both cause and effect levels; fixed the key pathway of metabolic changes, and further, the core regulatory network was constructed, thus revealing its inherent law [13, 14].

Soil salinization is very common in the Northeast China. It seriously affects the normal agricultural production and development. Therefore, the key to solve this problem is to identify salt-tolerant plants and to study the salt-stress-response mechanism in plants. *P. grandiflorus* is a traditional Chinese medicine, which is widely cultivated in the Northeast China and widely sold globally as a homologous plant. It is important to study the salt and alkali tolerance by this herbaceous plant to guide its cultivation and increase yield. Moreover, anti-inflammatory and expectorant properties in *P. grandiflorus* are mainly attributed to pentacyclic triterpenoid saponins. Among them, Platycodin D is present in high amounts. Platycodin D is one of the most abundant bioactive components in *P. grandiflorus*, which is prescribed as an index component by Chinese pharmacopoeia (2020). However, the effect of salt stress on the accumulation of Platycodin D in *P. grandiflorus* is not studied in detail. 2,3-oxidized squalene is the direct precursor of Platycodin D, which is mainly synthesized by the mevalonic acid pathway (MVA). Farnesyl pyrophosphate (Farnesyl-pp) is synthesized from isopentenyl diphosphate [15] and dimethylallyl pyrophosphate (DMAPP) in the presence of enzyme farnesyl pyrophosphate synthetase (FPP) [14, 16]. Squalene 2,3-oxide is synthesized by squalene synthetase (SS) and squalene epoxidase (SE). Subsequently, triterpene saponins are synthesized from 2,3-oxidized squalene through squalene oxidation cyclase, cytochrome P450 monooxygenase, and uridine diphosphate glycosyltransferase.

The aim of this study was to analyze the comprehensive responses of *P. grandiflorus* to salt stress. To study the molecular regulatory mechanisms under salt stress, we analyzed the related genes, metabolites, and important metabolic pathways via combined transcriptome and metabolome analyses. This study laid a foundation for further studies on the effect of salt stress and mechanism of salt tolerance in *P. grandiflorus* and provided new insights for further improving the salt tolerance and increasing the content of Platycodin D in *P. grandiflorus*.

## Results

### The growth and physiological changes in *P. grandifloras* under salt stress

**Fig. 1:**
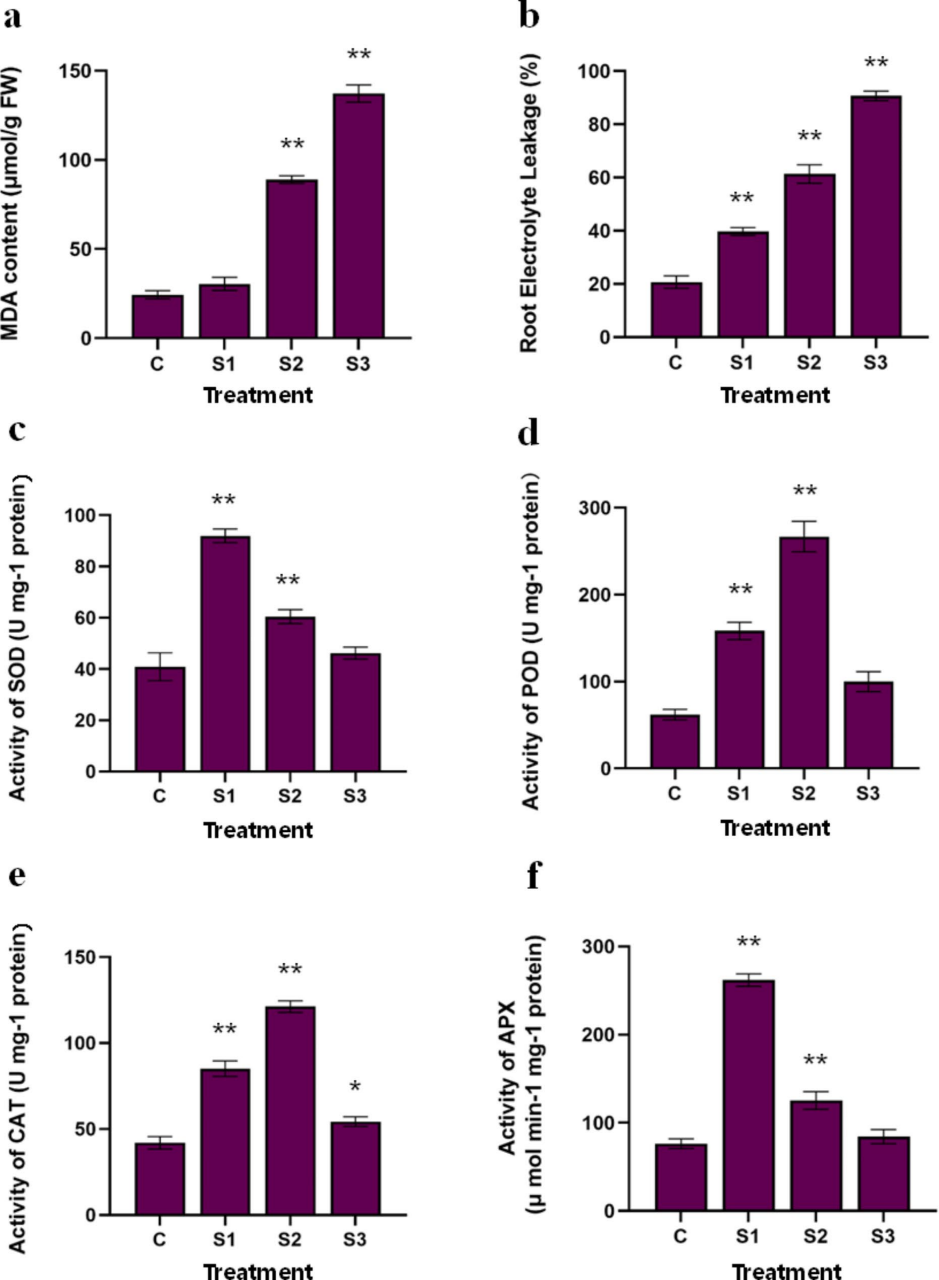
Effects of salt stress on physiological indexes of *Platycodon grandiflorus*. (**a**) MDA content; (**b**) Root electrolyte leakage %; (**c**) SOD activity; (**d**) POD activity; (**e**) CAT activity; and (**f**) APX activity. (* indicates significant difference at 0.05 level between salt stress group and control group; ** indicates significant difference at 0.01 level between salt stress group and control group. The same below.)

To study the physiological changes in *P. grandiflorus* under salt stress, the growth and physiological characteristics of *P. grandiflorus* under different concentrations of NaCl were recorded in C, S1, S2, and S3 groups. With the increase in salt concentration, the MDA content in S3 treatment was nearly 5.6-fold higher than that in the control group (Fig. 1a). Similarly, EL increased with the increase in salt concentration, and it increased by 4.4-fold in S3 compared with the control group (Fig. 1b).

The activities of SOD, POD, CAT, and APX were altered by salt stress (Fig. 1c–f). Compared with the control group, the SOD activity of *P. grandiflorus* root slightly increased under salt stress (Fig. 1c), reaching the peak in S1, which was nearly 1.13-fold higher than that in the control group. However, when the salt concentration further increased, the SOD activity decreased. Salt stress enhanced the activity of POD (Fig. 1d), reaching the maximum in S2, which was nearly 1.61-fold higher than that in the control group. The SOD activity in S3 decreased; however, it was higher than that in the control group. Salt stress altered CAT activity compared with the control (Fig. 1e). The activity of CAT was the same as that of POD, reached the maximum at S2, increased 1.29-fold compared with the control group, and decreased in S3 but was insignificantly higher than that in the control group. The change in APX activity under salt stress was the same as that in SOD activity (Fig. 1f), reaching the peak at S1, which was nearly 1.10-fold higher than that in the control group. When the concentration of salt further increased, APX activity exhibited a downward trend. These results indicated that SOD, POD, CAT, and APX were involved in the response of *P. grandiflorus* to salt stress.

**Fig. 2:**
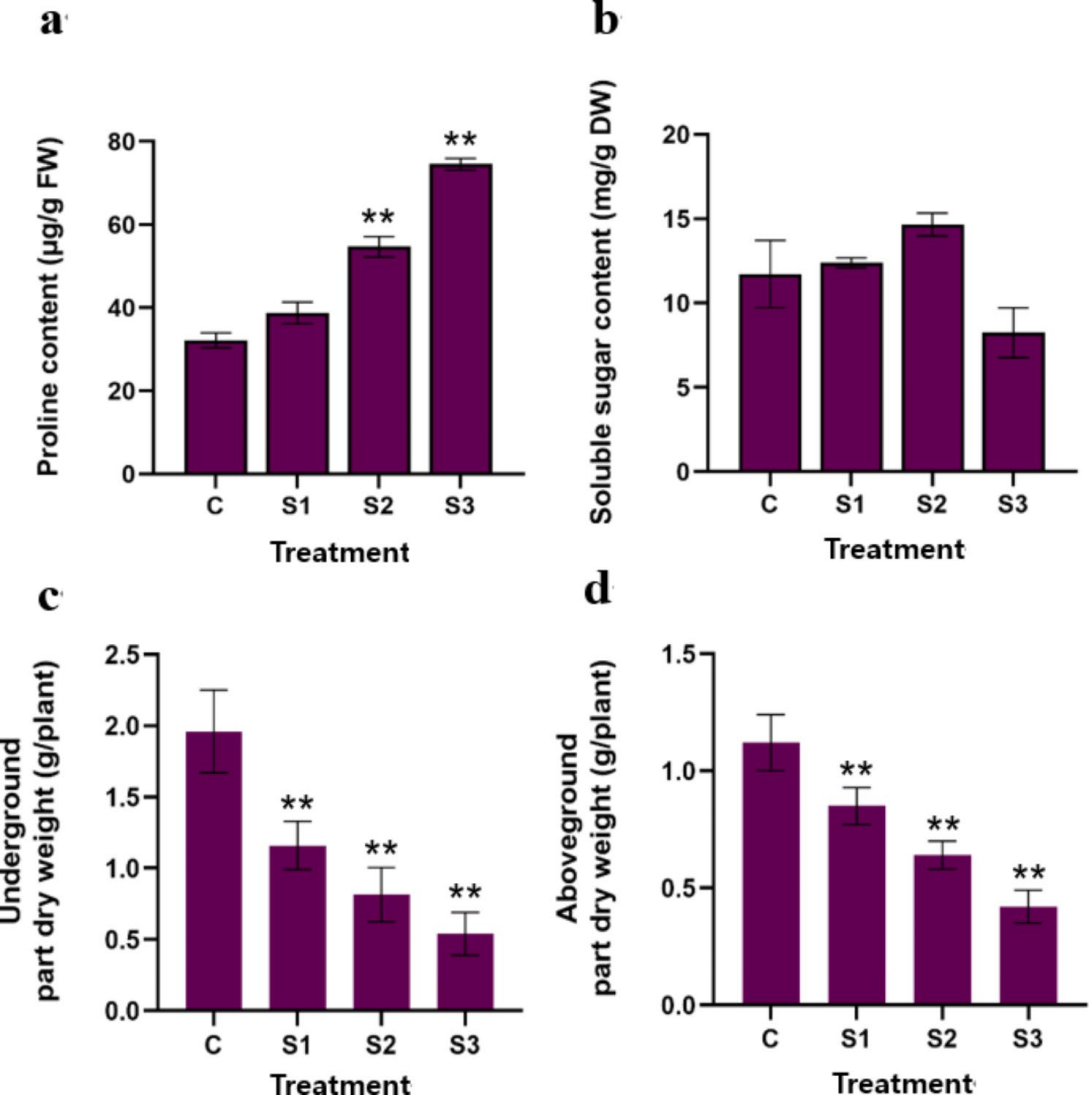
Effects of salt stress on the osmotic substances and biomass in *P. grandiflorus*. (**a**) proline content (**b**) soluble sugar content(**c**) underground part dry weight (**d**) aboveground part dry weight

The proline content in the roots of *P. grandiflorus* increased under salt stress (Fig. 2a). It exhibited slight increase under mild salt stress but significant increase under medium and severe salt stress. Compared with the control group, the content of proline was the maximum in S3 and increased by 131.79%. The content of soluble sugars in the roots of *P. grandiflorus* increased first and then decreased under salt stress (Fig. 2b). It increased by 5.63% and 25.06%, respectively, in S1 and S2 and decreased by 29.75% in S3 compared with the control group. The effect of salt stress on the growth of *P. grandiflorus* was evaluated. Salt stress reduced the dry weight of roots and aboveground parts of *P. grandiflorus* (Fig. 2c). The dry weight of roots significantly decreased with the increase in salt concentration and decreased by 72.45% in S3 compared with the control (Fig. 2d). The change in the dry weight of aboveground part exhibited the same trend, which was decreased by 62.5% in S3 compared with the control. These results indicated that salt stress has a significant impact on the growth of *P. grandiflorus*. based on these results, we combined transcriptomics and metabolomics to explore the mechanism of response of *P. grandiflorus* to salt stress.

### Transcriptome analysis and read assembly overview

To reveal the mechanism of salt tolerance in *P. grandiflorus* under salt stress, transcriptome analysis was performed using RNA-Seq method. A total of 24 cDNA libraries were constructed and sequenced. They represented four different groups of treatments, each with 6 replicates. A total of 513.9 million high-quality clean reads were obtained from the 24 cDNA libraries. All cDNA libraries had Q20 > 97.60%, Q30 > 93.35%, and GC content of approximately 43% (Supplementary Table 1). The clean reads from the orris were reassembled to obtain 270,489 transcripts with an average allele size of 1129 bp and an N50 allele size of 1839 bp (Supplementary Table 2).

Functional annotations of all single genes were annotated to the KEGG, NR, Swiss-Prot, NT, KOG, GO, and Pfam databases (Supplementary Table 3). In *P. grandiflorus*, 11,094 single genes (100%) were annotated in the databases. A total of 52,850 (48.00%) transcripts exhibited significant matches in the NR database, whereas 19,896 (18.07%), 40,996 (37.23%), 36,959 (33.57%), 17,870 (16.23%), 41,755 (37.92%), and 41,761 (37.93%) single genes had significant matches in the KEGG, Swiss-Prot, NT, KOG, GO, and Pfam databases, respectively. The transcript sequences of *P. grandiflorus*had 29.00% (*Quercus suber*), 7.10% (*Camellia sinensis)*, 5.80% *(Cynara cardunculus)*, 5.70% (*Vitis vinifera*), and 4.40% (*Actinidia chinensis*) similarity with those of the related species (Supplementary Fig. 1).

GO is a comprehensive database describing gene functions, which can be divided into biological process, cellular component, and molecular function. In *P. grandiflorus*, 41,755 transcripts were assigned to these three groups of GO terms (biological process 83,528, cellular component 37,128, and molecular function 47,746). Cell process, metabolic process, and biological regulation were the most abundant terms in biological process category. Anatomical entities, intracellular components, and protein containing complexes were the most important subcategories in cell components. The most representative terms in molecular function were binding and catalytic activity (Supplementary Fig. 2). Furthermore, the annotated sequences were reflected in the KEGG pathway. In *P. grandiflorus*, 19,896 single genes were assigned to 296 KEGG pathways. Notably, 132 sequences were annotated for the metabolism of terpenoids. These annotation results laid a foundation for further study on the metabolic pathway of *P. grandiflorus*.

### Analysis of differential gene expression in *P. grandiflorus*

**Fig. 3:**
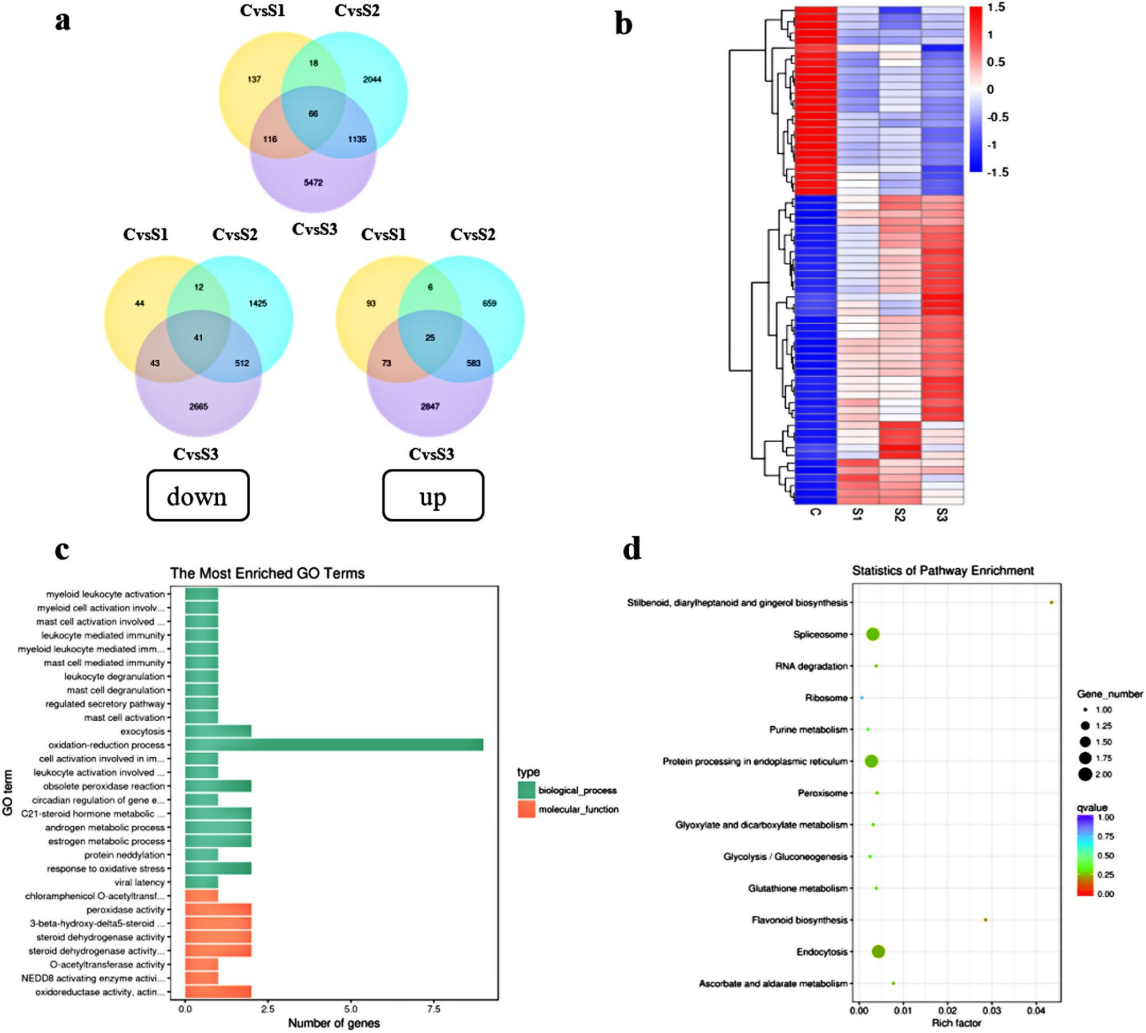
Transcriptome analysis of *P. grandiflorus* was performed by high-throughput sequencing. (**a**) Venn diagram showing the number of DEGs among different treatments and the only upregulated and downregulated DEGs; (**b**) Hierarchical analysis of the overlapping 66 DEGs at different concentrations of salt stress. (In the graph, the horizontal coordinates represent different experimental groups, the vertical coordinates represent the corresponding differentially expressed genes in this group, the color blocks in different positions represent the relative expression amount of genes in the corresponding positions, and the red color represents the high expression of this gene, blue indicates low expression of this gene.) (**c**) GO pathway enrichment analysis based on the 66 overlapping DEGs; and (**d**) KEGG pathway analysis based on the 66 overlapping DEGs

A total of 8,988 DEGs were obtained. Venn plots revealed that 66 of them (25 upregulated and 41 downregulated) overlapped in all the three treatment. They all exhibited different expression patterns (Supplementary Tables 4 and Fig. 3a and b). These results indicated that DEGs were expressed in the roots of *P. grandiflorus* under three different concentrations of salt. To determine the main functional terms of these 66 overlapping DEGs, GO enrichment analysis was performed. Analysis of GO terms revealed that most of these 66 DEGs were enriched in biological processes, particularly in oxidation–reduction processes (Fig. 3c). KEGG pathway analysis revealed that most of the 66 overlapping DEGs were significantly enriched in spliceosome, protein processing in the endoplasmic reticulum, and biosynthetic pathways of endocytosis (Fig. 3d).

Salt stress can lead to the rapid accumulation of ROS, destroy the redox balance of cells and cell membrane, and damage macromolecules. Major antioxidase-related genes play an important role in ROS detoxification systems. Here, we identified 5 different antioxidase-related genes in all DEGs (Supplementary Table 5), including two APX, one SOD, one CAT, and one POD. Among the 24 DEGs, two SOD genes (Cluster-44689.29455 and Cluster-44689.26870) and one CAT gene (Cluster-44689.32480) were upregulated in S3; the other POD and APX genes were upregulated and downregulated to various degrees in the three salt treatment groups.

The late embryonic abundant (LEA) protein plays a key role in reducing the effect of ROS and protecting the integrity of cell membrane. In addition, LEA protein has been identified to cope with water shortage response caused by drought, salinity, and osmotic stress in many plants. Among the sequenced DEGs, we identified four LEA genes. One LEA gene (Cluster-44689.28642) was upregulated in *P. grandifloras* under S2; one LEA gene (Cluster-44689.1424) was downregulated in *P. grandiflorus* under S3, and the remaining two LEA genes (Cluster-44689.28642 and Cluster-44689.23442) were upregulated in S3.

### Metabolomics analysis of *P. grandiflorus* under salt stress

**Fig. 4:**
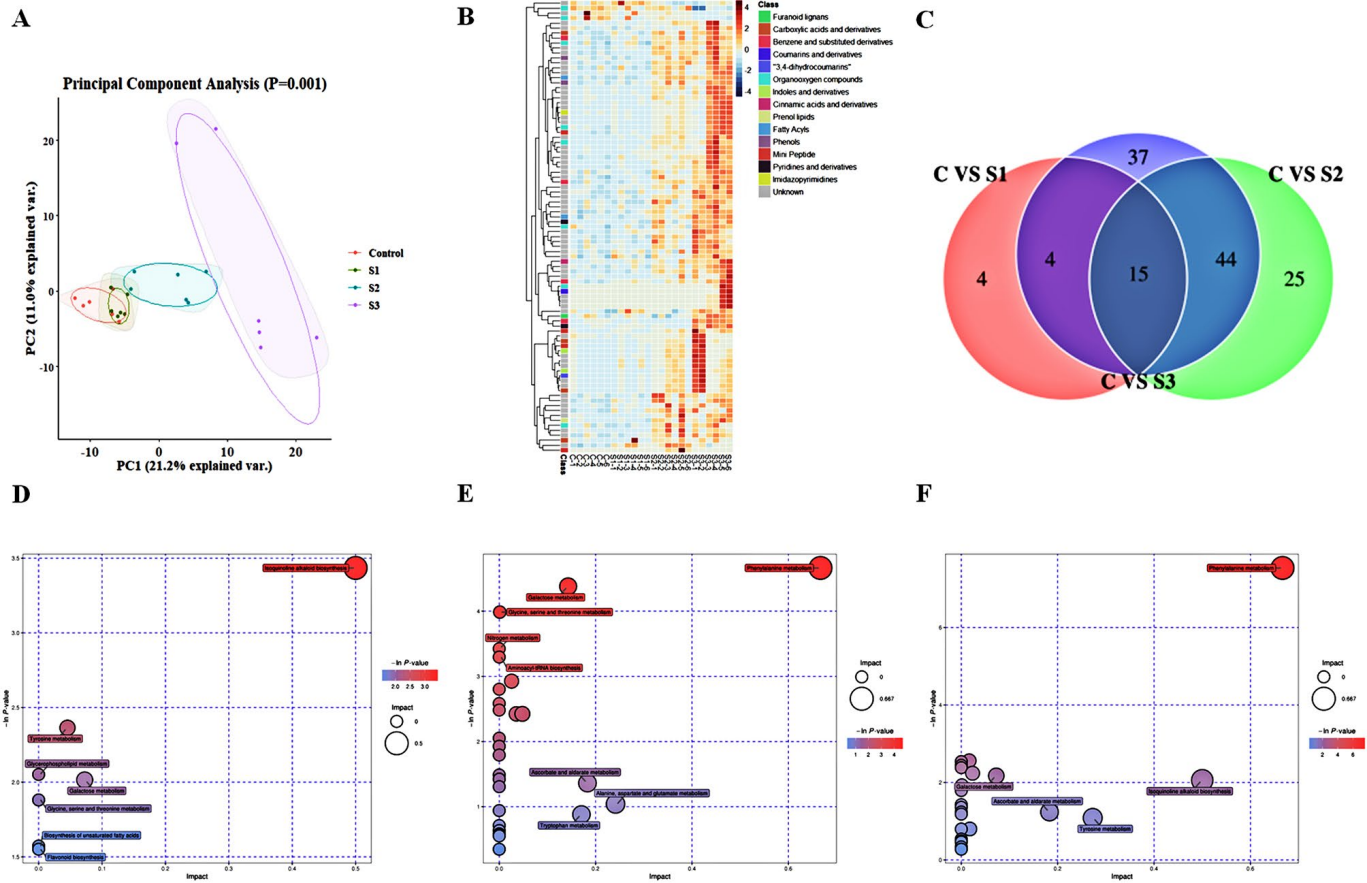
Metabolomic analysis of differences among different salt stress treatments. (**a**) The PCA of all metabolites expressed in *P. grandiflorus* under different levels of salt stress; (**b**) Heat map of metabolite enrichment under salt stress; (**c**) Venn diagram showing the number of differentially abundant metabolites among different treatments; KEGG pathway analysis of differentially abundant metabolites in (**d**) S1, (**e**) S2, and (**f**) S3 compared with the control

Before analyzing the differentially abundant metabolites, we conducted PCA to detect the degree of variation among and within groups. PCA could clearly distinguish 24 samples, accounting for 32.2% of the total variability (Fig. 4a). It can be seen from the figure that the aggregation between the four treatment groups showed significant differences, among which the differences between the control group and S1, S2 and S3 increased in turn. The six biological replicates in each group clustered together; the six biological replicates in S3 were the most dispersed and most significant, followed by S2 and S1. The replicates in the control group were the least significantly dispersed.

We determined a total of 428differentially abundant metabolites and plotted a heat map to indicate the abundance of metabolites at different salt concentrations (Supplementary Figs. 3–5). A series of changes in metabolites occurred in *P. grandiflorus* under salt stress. The tested metabolites were divided into 15 classes (Fig. 4b); the first three classes were furan lignin, carboxylic acids and their derivatives, and benzene and its substituted derivatives. According to the heat map, the metabolites in S1 exhibited an overall upward trend. The differentially abundant metabolites under moderate salt stress S2 were significantly different from those in the control, and more metabolites were upregulated in S2. The highest number of metabolites were upregulated in S3, and their levels were higher than those in S2.

In addition, from the 428 differentially abundant metabolites (DAMs), 23 DAMs (18 upregulated and 5 downregulated) were identified in CvsS1 according to the screening condition of VIP > 1 and p value < 0.05 (Supplementary Table 6). In CvsS2, 84 DAMs (58 upregulated and 26 downregulated) were identified (Supplementary Table 7). In CvsS3, 100 DAMs (88 upregulated and 12 downregulated) were identified (Supplementary Table 8). Overall, 15 overlapping DAMs, such as (s)-p-mentha-1,8-dien-7-ol, 9s, 10R-Epoxy-6Z-octadecene, anethole, and choline, were shared among the three treatment groups (Fig. 4c). Compared with the control group, the content of differentially abundant metabolites increased with the increase in salt concentration, and the differentially abundant metabolites were mostly upregulated.

KEGG enrichment analysis revealed that the metabolic pathways were classified and distributed differently under different salt stress treatments. In CvsS1, the 7 DAMs were classified and assigned to 7 metabolic pathways. They were mainly primary metabolic pathways. The most significantly enriched pathway was isoquinoline organism biosynthesis (ko00950) (Fig. 4d). As the salt stress increased, 44 metabolites were assigned to 27 metabolic pathways in CvsS2; the most significantly enriched pathway was phenylalanine metabolism (ko00360), followed by galactose metabolism (ko00052) and glycine, serine, and threonine metabolism (ko00260) (Fig. 4e). Under severe salt stress, 35 metabolites were assigned to 25 metabolic pathways (Fig. 4f). The most significantly enriched pathway was phenylalanine metabolism (ko00360). Phenylalanine metabolism mainly participates in the secondary metabolism, such as flavonoid and lignin metabolism pathways. Some metabolites, such as L-phenylalanine, are precursors of flavonoid biosynthesis. These compounds are natural secondary metabolites and play an important role in plant defense. Lignin is an important substance present in plant cell wall, which can enhance the resistance of plants to biotic or abiotic stress. Therefore, we next combined transcriptomics and metabolomics to analyze the lignin metabolism pathways and other secondary metabolism under salt stress.

**Fig. 5:**
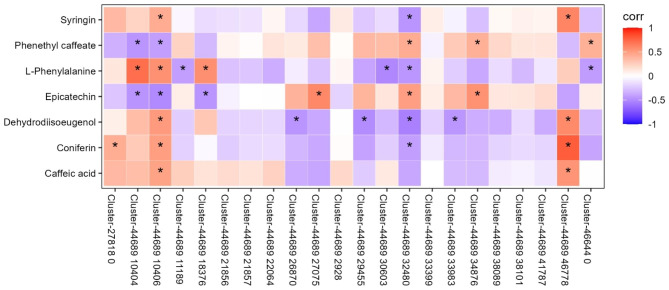
Correlation analysis of DEGs and differentially abundant metabolites under different salt stress treatments. A total of 22 DEGs and 7 differentially abundant metabolites were used for correlation matrix analysis. * p value < 0.05 of differentially abundant metabolites and genes

Through the analysis of DEGs and differentially abundant metabolites among different treatments, 101 DEGs and 22 differentially abundant metabolites among different treatments were identified in the annotation database (Supplementary Table 9). The correlation coefficient (Corr) and p value of the differentially abundant metabolites and DEGs were measured using Spearman algorithm, which revealed that 99 DEGs had significant correlation with the 22 DAMs (Supplementary Fig. 6). Among them, 7 key metabolites and 22 oxidoreductase related genes in response to salt stress in *P. grandiflorus* were screened and further correlation analysis was conducted (Fig. 5). Analysis showed that Caffeic acid, Syringin, Coniferin, and Dehydrodiisoeugenol were all significantly positively correlated with POD-related genes (Cluster-44689.10406, Cluster-44689.46778); Syringin, Coniferin, Dehydrodiisoeugenol, L-Phenylalanine were negatively correlated with CAT-related genes (Cluster-44689.32480), while Epicatechin and Phenethyl caffeate were negatively correlated with CAT-related genes (Cluster-44689.32480). Dehydrodiisoeugenol was significantly negatively correlated with SOD-related genes (Cluster-44689.26870, Cluster-44689.29455). Among them, Caffeic acid, Syringin, Coniferin and L-Phenylalanine are the key metabolites in lignin biosynthesis pathway. These results suggest that lignin compounds are significantly related to antioxidant enzymes and participate in the salt stress response of *P. grandiflorus*, and show some stress resistance to adapt to salt stress. In addition, other oxidoreductase genes were positively or negatively correlated with most of the metabolites with different abundances.

Figure 5 Correlation analysis of DEGs and differentially abundant metabolites under different salt stress treatments. A total of 22 DEGs and 7 differentially abundant metabolites were used for correlation matrix analysis. * p value < 0.05 of differentially abundant metabolites and genes.

### Comprehensive analysis of genes and metabolites associated with the biosynthesis of lignin compounds after salt stress treatment

**Fig. 6:**
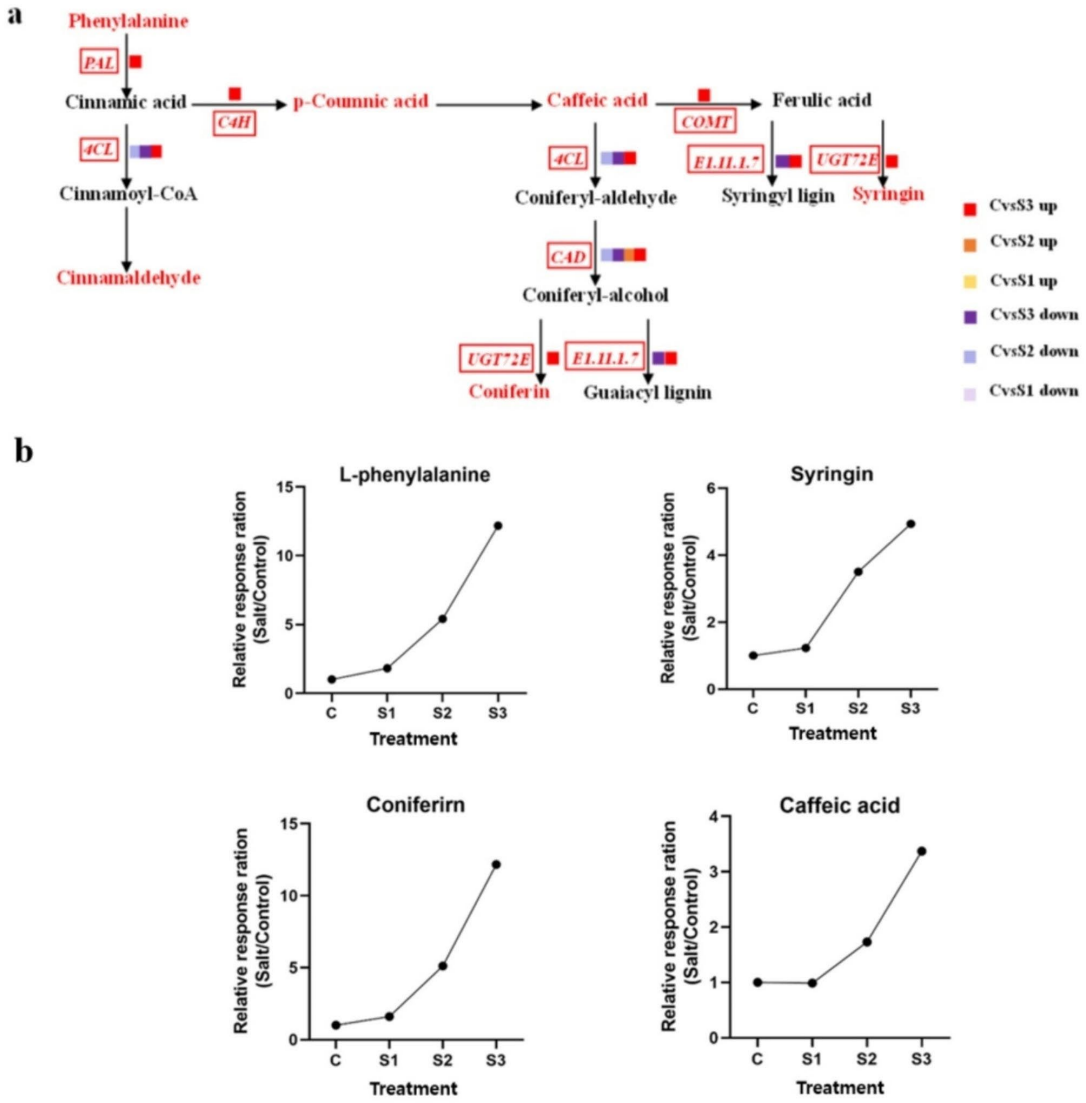
Lignin-related pathway in *P. grandifloras* under different salt stress treatments. (**a**) The proposed metabolic pathway is based on KEGG pathway map: Ko00940 Phenylpropanoid biosynthesis (http://www.kegg.jp/kegg/kegg1.html). The metabolites in red color were the differentially abundant metabolites detected in this study. Metabolites in black color were not detected. The red italics and small icons represent genes, whereas purple color represents downregulation and red represents upregulation; (**b**) Salt-induced lignin concentration relatively changed compared with the control

Genes and metabolites in the biosynthesis pathway of lignin-like compounds were affected after different salt stress treatments. A total of 11 genes involved in the biosynthesis of lignin-like compounds with a total of 151 transcripts were up- or downregulated in CvsS2 and CvsS3, and the highest number of up- and downregulated transcripts were in CvsS3 (Fig. 6a and Supplementary Table 10). They were *PAL* (phenylalanine ammonia lyase), *4CL* (4-coumarin-CoA ligase), *C4H* (trans-cinnamic acid 4-monooxygenase), *CCR* (cinnamoyl-CoA reductase), *CAD* (cinnamyl alcohol dehydrogenase), *POD* (peroxidase), and *UGT* (coniferyl-alcohol glucosyltransferase).

The ratio of the relative concentration of metabolites to that in the control under different salt concentrations changed by varying degrees, which indicated that the metabolites in *P. grandiflorus* changed significantly under salt stress. Four significantly altered metabolites were L-phenylalanine, cinnamic acid, syringin, and coniferin (Fig. 6b). Compared with the control, these metabolites were not significantly changed in CvsS1; however, with the increasing salt stress, these metabolite levels were significantly increased compared with the control. The relative amount of L-phenylalanine and coniferin increased by almost 12-fold and that of caffeic acid and syringin increased by almost 3.0 and 4.0-folds, respectively, under severe salt stress compared with the control. The expression of DEGs related to these metabolites was altered. *PAL* (Cluster-44689.41787, Cluster-44689.33983, and Cluster-44689.38089) and *C4H* (Cluster-44689.40920, Cluster-44689.46702, and Cluster-44689.35908) were the key enzyme genes in the biosynthetic pathway of caffeic acid; all of them were upregulated, and the genes related to the degradation of caffeic acid were significantly downregulated in CvsS3 (Cluster-44689.16721, Cluster-44689.16723, Cluster-44689.16722, and Cluster-44689.46778). This may explain the significant increase in the content of caffeic acid. POD is a heme-containing enzyme in plants that targets the cell wall or vesicle secretion pathway and is defined as a class-III plant peroxidase [17], which was up-regulated in CvsS2, but there was no significant difference in POD between CvsS3 and control. *CAD* (Cluster- 44689.11189, Cluster-44689.45486, Cluster-44689.2148, Cluster-44689.33731, Cluster-44689.35058, Cluster-44689.32157, Cluster-44689.17546, Cluster-44689.32201, and Cluster-44689.32203) and *UGT* (Cluster-44689.47177) that catalyzed the reduction of pinoresinol and mustard alcohol respectively were significantly upregulated in CvsS2 and CvsS3. This may have significantly increased syringinin content.

### Comprehensive analysis of genes and metabolites associated with triterpene saponin biosynthesis after salt stress treatment

**Fig. 7:**
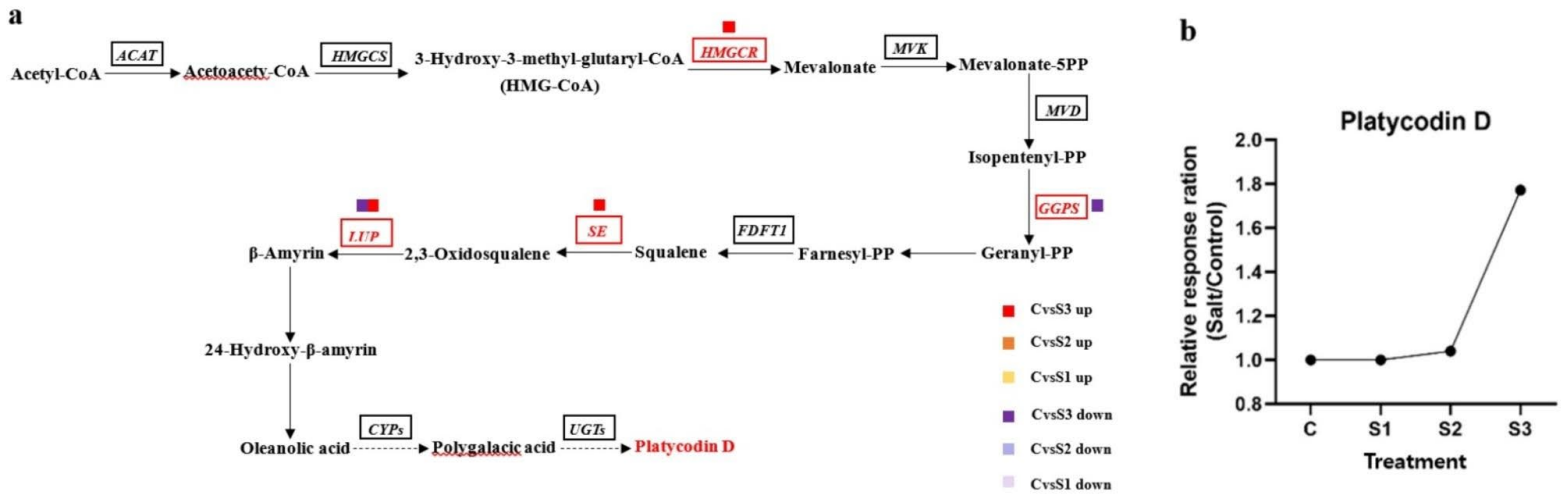
Platycodin D-related synthesis pathway under salt stress. (**a**) The proposed metabolic pathway is based on the literature and network. The metabolites in red color were the differentially abundant metabolites detected in this study. The metabolites in black color were not detected. The red italics and small icons represent genes, whereas purple color represents downregulation and red represents upregulation; (**b**) Salt-induced Platycodin D concentration relatively changed compared with the control

Terpenoids are the main secondary metabolites in *P. grandiflorus*. The initial pathway is the MVA pathway. The first step of the MVA pathway is the conversion of acetyl-CoA into HMG-CoA by AACT and 3-hydroxy-3-methylglutaryl-coenzyme A synthase. The enzyme 3-hydroxy-3-methylglutaryl-coenzyme A reductase converts HMG-CoA to MVA. With mevalonate kinase, MVA is converted into mevalonate 5-phosphate. Furthermore, with 5-phosphomevalonate kinase, mevalonate 5-diphosphate (MVAPP) is synthesized. MVAPP is converted into isopentenyl diphosphate [15] in the presence of mevalonate diphosphate decarboxylase. Finally, 2, 3-oxidosqualene, the direct precursor of triterpenoid saponins, is synthesized. Some key enzymes involved in the triterpenoid biosynthetic pathway were identified in the plant (Fig. 7).

Platycodin D is the main active component of *P. grandiflorus*. It is a pentacyclic triterpene saponin and is mainly synthesized by the MVA pathway. However, the role of MVA pathway in regulating triterpene saponin biosynthesis under salt stress has not been clearly understood. In the study, based on the ratios of metabolite concentrations in the salt-stress treatments and control, the relative content of Platycodin D increased significantly with the increase in salt stress. In S3, it increased by 1.77-fold compared with the control. A total of 10 genes involved in the MVA pathway of terpenoid skeleton biosynthesis were identified via homology search (Fig. 7 and Supplemental Table 11). The conversion pathway of triterpenoid saponins in *P. grandiflorus* was predicted based on the triterpenoid backbone [18]. The terpene backbone is enriched with 10 DEGs; the key enzyme gene in the generation of mevalonate from 3-hydroxy-3-methylglutaryl coenzyme A, namely, *HMGCR* (hydroxymethylglutaryl-CoA reductase) was upregulated in the upstream part of the triterpenoid saponin biosynthetic pathway. Two transcripts related to *GGPS* (geranylgeranyl diphosphate synthase, type II) were downregulated. *GGPS* is the key enzyme gene that catalyzes the formation of geranyl-PP from isopentenyl-PP. One transcript related to *SE* (squalene monooxygenase) was upregulated. *SE* is the enzyme gene responsible for the first oxidation step in the biosynthesis of phytosterols and triterpenoids. This enzyme catalyzes the double-bonded epoxidation of squalene to generate 2,3-oxidized squalene (Benjaminlt and Hochberg 1995). In the downstream part, *LUP4* (beta-amyrin synthase β-AS), in which one transcript is downregulated and five transcripts are upregulated, is an intermediate in the synthesis of oleanolic acid pentacyclic triterpenes from 2,3-oxidized squalene in the triterpenoid saponin synthesis pathway, which in turn generates oryzae saponin. It is a key enzyme gene that catalyzes the production of β-amyrin from farnesyl-PP (FPP). *LUP* was upregulated under moderate and severe salt stresses, and the number of upregulated genes was the highest under severe salt stress, which promoted the synthesis of oleanolic acid pentacyclic triterpene saponins. Therefore, the content of Platycodin D increased with the increase in salt concentration, especially under severe salt stress.

Figure 7 Platycodin D-related synthesis pathway under salt stress. (**a**)The proposed metabolic pathway is based on KEGG pathway map: Ko00900 Terpenoid backbone biosynthesis (http://www.kegg.jp/kegg/kegg1.html). The metabolites in red color were the differentially abundant metabolites detected in this study. The metabolites in black color were not detected. The red italics and small icons represent genes, whereas purple color represents downregulation and red represents upregulation; (**b**) Salt-induced Platycodin D concentration relatively changed compared with the control.

## Discussion

**Fig. 8:**
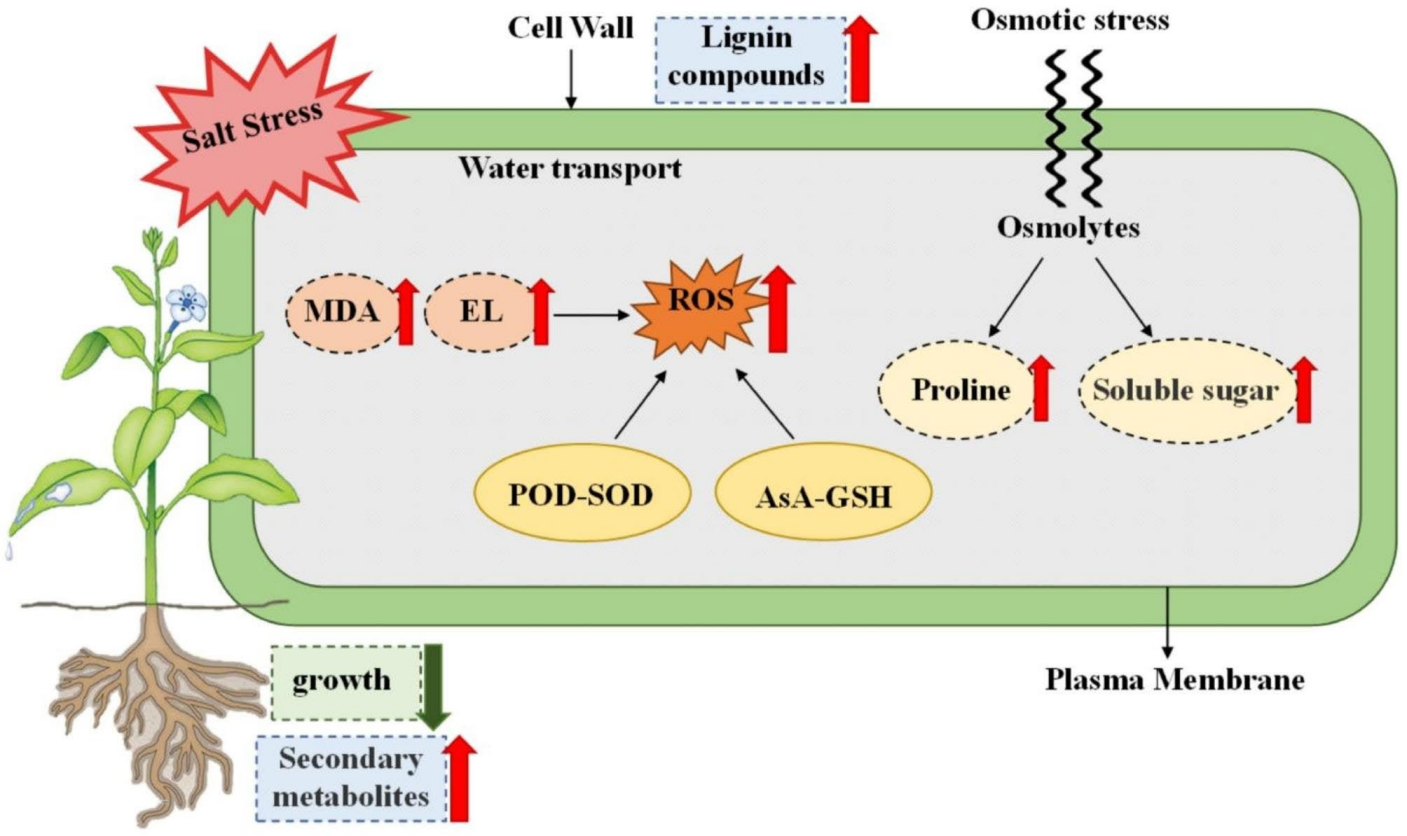
The possible mechanisms of response of *P. grandifloras* to salt stress

Studies demonstrated that salt stress forces plants to produce significant amount of ROS [5], which leads to lipid peroxidation, denaturation, membrane damage, and other effects in plants and has inhibitory effect on plant growth (Reddy et al., 2004). With the increase in salt content during the growth phase, plant leaf area gradually stops growing, and the fresh and dry weights of leaves and roots exhibit a downward trend. Our study demonstrated that *P. grandifloras* may respond to salt stress through the following mechanisms (Fig. 8). With the increase in salt concentration, the dry weight of *P. grandiflorus* gradually decreased; the dry weight of the underground part decreased by more extent compared with that of the aboveground part. This indicated that the growth inhibitory effect of salt was greater on the underground part than on the aboveground part of *P. grandiflorus*. The accumulation of ROS can damage the membrane, leading to increased MDA and EL. MAD is one of the most important products of membrane lipid peroxidation, which can aggravate membrane damage (Blokhina et al., 2003). In this study, the content of MDA increased with the increased salt stress, which indicated that salt stress induced excessive ROS production and plasma membrane damage. Plants have developed some mechanisms to mitigate these harmful effects. These mechanisms include antioxidant defense system composed of enzymatic and nonenzymatic antioxidants, which can maintain the balance between ROS production and degradation. Increased antioxidant enzyme activity is a universal adaptive response to salt stress in plants (Sarvajeet and Narendra 2010). In this study, the activity of SOD, POD, CAT, and APX increased significantly under mild salt stress (S1 200 mmol/L NaCl), which indicated that different antioxidant enzymes exhibited an integrated effect in scavenging ROS. SOD is the most important line of defense in the antioxidant enzyme defense system, especially against superoxide anion radicals (O_2_^−^) (Gill and Tuteja 2010; Zelko et al., 2002). In the present study, genes regulating SOD synthesis (Cluster-44689.26870 and Cluster-44689.29455) were upregulated under salt stress. The main product of SOD is a toxic lipid peroxide, i.e., hydrogen peroxide (H_2_O_2_), which can be removed by POD and CAT (Sabine et al., 2004). Under certain stress, the activities of POD and CAT increase, which may play a protective role in anti-oxidation in plants. POD plays a key role in removing H_2_O_2_ and MDA, thereby protecting cell membrane integrity (Hojati et al., 2011). Our results of increase in POD activity are consistent with those of Fan et al. (2009) and Hojati et al. (2011), indicating that the POD–SOD system had an important protective effect in plants. APX is part of the AsA-GSH cycle and is effective in preventing ROS. APX levels increased significantly under mild salt stress, indicating that APX is involved in the antioxidant activity of AsA-GSH cycle under salt stress. Proline and soluble sugars are important intracellular osmotic pressure regulators. Proline accumulation is proportional to the external osmotic pressure of the cell. Its main function is to protect the structure of the cell and maintain osmotic balance within cells by regulating water (Hasegawa, et al. 2000). In this study, the contents of proline and soluble sugars increased with the increasing salt stress. This is consistent with the studies on *Lonicera japonica* and *Olea europaea* under salt stress [15, 19]. Moreover, the key enzyme genes regulating proline synthesis (Cluster-44689.39488, Cluster-44689.40382, Cluster 44689.41279, Cluster-44689.36855, Cluster-44689.30781, and Cluster-44689.29410) were upregulated. These results indicated that salt stress increased ROS in *P. grandiflorus* but activated some SOD and proline synthase genes to increase SOD activity and proline content, which could alleviate the oxidative damage and damage to intracellular balance caused by salt stress. At the same time, we observed that under severe salt stress, the enzyme activity decreased and MDA content still significantly increased. This indicated that very high salt concentration would destroy the function of enzymes, thereby significantly reducing the scavenging capacity of ROS, intensifying the oxidative damage of membrane lipids, and thus causing irreversible damage to the growth and development of *P. grandiflorus*.

Salt stress not only has a certain impact on plant growth but also causes greater osmotic stress and oxidative damage to plants [20]. Therefore, plants may develop a complex salt-resistant mechanism to mitigate salt stress damage. Transcriptomic analysis revealed that photosynthesis in the salt-tolerant genotypes of *Phaseolus vulgaris* L. increased under saline conditions, and metabolite analysis using GC–MS revealed increased carbohydrate metabolism, increased sugar content, and better amino acid metabolism in such salt-tolerant genotypes,; this successfully prevented the accumulation of Na^+^ in leaves and roots, thus alleviating the adverse effects of salt stress [21]. Furthermore, studies have reported that hub genes responsive to salt stress (*TLP1, WRKY6, ATHB7*, and *EF109*) play a role in tolerance to adverse conditions and protect cotton crops from the effects of salt stress. The protection of these hub genes can reduce the loss of crop productivity and provide resistance under adverse conditions [22]. In the study, among the 25 shared upregulated genes in each treatment, two genes (Cluster-44689.34876 and Cluster-44689.32268) were for heat shock 70-kDa proteins (Hsp70). Heat shock proteins (HSPs) have biological antioxidant activity in cells and enhance the resistance of cells to stress. Under salt stress, the level of oxygen free radicals in cells increases, and they significantly affect the permeability and fluidity of biofilm, thus destructing the organelles and subcellular organs. At the same time, the antistress system of plants produces HSPs to protect cells from damage. They can inhibit adenine dinucleotide coenzyme (NADPH), a key enzyme that produces oxygen free radicals, and inhibit the increase in the levels of oxygen free radicals through feedback. In addition, HSPs can help the antistress system of the body in eliminating oxygen free radicals by directly releasing and increasing the level of endogenous peroxidase such as SOD, thus playing a role in cell protection. In addition, another APX enzyme gene (Cluster-44689.27075) was upregulated to adapt to salt stress. Therefore, under salt stress, *P. grandiflorus* could not only inhibit the increase in the levels of oxygen free radicals by regulating the activity of antioxidant enzymes but also enhance the resistance of cells to salt stress by upregulating the Hsp70 gene to stabilize the cell structure and reduce the content of ROS.

The metabolome data revealed that the differentially abundant metabolites in *P. grandiflorus* were mainly concentrated inisoquinoline alkaloid biosynthesis under mild salt stress. The increase in NADPH concentration in stressed plants leads to the increase of biosynthesis rate, which leads to the increase of alkaloid concentration in plants under various stress conditions [23]. The increase in stress-related alkaloid content may be due to the well-known stress-related passive transfer and increased enzymatic capacity. On the other hand, isoquinoline alkaloids are derived from the L-phenylalanine and tyrosine systems, and the L-phenylalanine and tyrosine contents increase under salt stress, which may also contribute to the biosynthesis of isoquinoline alkaloids. Under moderate and severe salt stresses, the differentially abundant metabolites were mainly concentrated in L-phenylalanine metabolism. The L-phenylalanine pathway is one of the most important pathways in secondary metabolism in plants. In the form of monomers or complexes, existence in specific tissues and organs of plants and plays an important role in morphogenesis, growth, and development in plants. In addition, it is involved in plant signaling in response to various stresses [24]. For example, it is involved in the synthesis of lignin compounds. Lignin is a component of the vasculature that makes the xylem mechanically supportive, transports water and nutrients, and participates in membrane lipid peroxidation in cells after salt stress. PAL is a key enzyme linking primary and L-phenylalanine metabolism. It is involved in secondary metabolic pathways in vascular plants. In addition, it is mainly involved in defense mechanisms and plays an important role in cell differentiation, lignification, and stress resistance in plants [25]. In this study, the key enzyme genes of PAL were upregulated under salt stress, and the content of lignin and other secondary products was increased. This suggested that *P. grandifloras* resists growth inhibition by salt stress via increasing the content of its secondary products in response to L-phenylalanine metabolism.

Combining the metabolomic and transcriptomic data, we obtained more information on some of the key metabolic pathways and related genes. Lignin is the main structural component of the cell wall of terrestrial higher plants and is the second most abundant plant polymer after cellulose. It is essential for mechanical support and water transport and plays a defensive role against stress [26], which can improve the stress resistance of plants [27]. In the present study, the synthesis of lignin-like compounds was accompanied by the changes in gene expression in response to salt stress; *PAL*, *C4H*, *UGT72E*, and *COMT* were upregulated, and *4CL* and *CAD* were both upregulated and downregulated. According to the differentially expressed metabolites, phenylalanine content in *P. grandiflorus* was increased after salt stress treatment. Phenylalanine can produce several precursors to synthesize lignin. The enzymes *PAL* and *C4H* play an important role in the lignin synthesis pathway. They are the early enzymes in the lignin synthesis pathway. PAL catalyzes the conversion of L-phenylalanine to cinnamic acid, which is the structural unit of lignin. Caffeic acid synthesis catalyzed by *PAL* and *C4H* was similarly increased. *UGT72E2*, the main GT involved in monophenolic glycosylation in plant roots [28], is a key enzyme gene for the further catalytic production of coniferin and the further production of syringin from sinapyl alcohol. Therefore, after salt stress treatment, with *UGT72E2* upregulation, coniferin and syringin content was increased in *P. grandiflorus*. *COMT* is a methyltransferase that methylates L-phenylalanine, a lignin precursor, and is a key enzyme gene in the lignin synthesis pathway. Thus, *COMT* is also a key enzyme gene for the production of coniferyl alcohol and thus sinapyl alcohol from caffeyl alcohol under salt stress [29, 30]. This study demonstrated that salt stress can regulate the synthesis of lignin compounds by changing the expression patterns of many enzymes involved in lignin biosynthesis. Under salt stress, the upregulation of lignin compounds can increase the lignification degree of plant cell wall, which can effectively prevent the internal ion absorption of cells and enhance the structural rigidity and firmness of conducting tissues [31] to improve salt tolerance in plants.

Many terpenoids with different structures are involved in the process of plant growth and development. They are important substances affecting plant physiological activities and interacting with environmental stress [32]. Among them, triterpene saponins are one of the major components in *P. grandiflorus*, and the representative active component is Platycodin D. To explore the molecular regulation mechanism of Platycodin D under salt stress, the transcription and expression of key enzymes in its metabolism were analyzed in this study. *HMGCR* genes involved in the MVA pathway were significantly upregulated in CvsS3. *GGPS* genes were significantly downregulated in CvsS3, and *SE* genes were upregulated in CvsS3 under salt stress in *P. grandiflorus*. In contrast, *LUP4* was downregulated in CvsS2 and upregulated in CvsS3. Among all DEGs involved in the synthesis of Platycodin D, *HMGCR* in the mid-upper portion is the rate-limiting enzyme of the MVA pathway [33], suggesting that higher concentration of salt in CvsS3 led to its upregulation to limit the production of subsequent products of the MVA pathway. However, its overexpression could not overcome the limitation of the MVA pathway. The key gene for terpene backbone biosynthesis, *GGPS*, controls the synthesis of geranyl diphosphate, which precedes the biosynthesis of sesquiterpenes and triterpenes (the saponin backbone biosynthetic pathway) [34]. Lower *GGPS* transcript levels reduce the production of terpene precursor, thereby affecting the downstream synthesis of terpenoids. However, the SE-catalyzed production of 2,3-oxidosqualene in the downstream fraction was instead upregulated under high salt stress, and 2,3-oxidosqualene is considered a common precursor of steroid saponins, triterpenoid saponins, and phytosterols [5, 35, 36]. In addition, *LUP4*, which catalyzes the production of β-amyrin from FPP in the downstream fraction, is also an important enzyme gene for the synthesis of oleanolic acid pentacyclic triterpenes from 2,3-oxidized squalene. It in turn generates orris saponins, is downregulated in response to moderate concentration of salt in CvsS2, and is upregulated again at high concentration of salt. However, some other genes *(HMGCR*, *GGPS*, and *SE*) spontaneously produce different levels of response with increasing salt stress to mitigate the damage caused by salt stress to guarantee the normal synthesis of Platycodin D. The specific regulatory effect of salt stress on the biosynthesis of Platycodin D remains unclear and further studies are ongoing.

## Conclusions

In summary, salt stress inhibited the growth of *P. grandiflorus* and reduced the dry weight of the above- and belowground parts of *P. grandiflorus*. MDA content and EL increased with the increase in salt stress. The ability of *P. grandiflorus* seedlings to adapt to salt stress was enhanced via upregulation of antioxidant system and osmotic adjustment. In addition,salt stress decreased the dry weight of roots but increased accumulation of some bioactive secondary metabolites in roots, such as lignin compounds and triterpene saponins. The lignin compounds are the main structural components of the cell wall, which may play a defensive role against salt stress. Salt stress significantly increased the accumulation of Platycodin D, the main effective component of *P. grandiflorus*. Our study provided the mechanism of response of *P. grandiflorus* to salt stress and biosynthesis of active ingredients in *P. grandiflorus* under salt stress. This may be useful to guide cultivation of *P. grandiflorus* and increase the active ingredient content.

## Materials and methods

### Plant cultivation and salt stress treatment

The study was conducted in Inner Mongolia Medical University greenhouse (40 ° 56’ N, 111 ° 48’ E, elevation 1230 m) in Hohhot, Inner Mongolia, Northwest China. As experimental material, 2-year-old seedlings of *P. grandiflorus* were used. The seedlings grown in the base were transplanted into a plastic pot (25-cm high and 17-cm diameter). The pot was filled with soil taken from the base. Soil physical and chemical properties and basic salt content were measured before planting. The soil pH, organic matter content, hydrolyzed nitrogen, available phosphorus, and available potassium were 7.7, 30.5 g/kg, 132.4 mg/kg, 27.5 mg/kg, and 247 mg/kg, respectively. Before beginning the experiment, the normal growth cultivation of the *P. grandiflorus* potted seedlings was performed to ensure the consistency of the experimental treatment.*P. grandifloras* seedlings with the same growth potential were selected for the salt stress treatment. The experiment included four treatments with six pots for each treatment (24 pots in total) and five seedlings in each pot. The treatments were as follows: control (C, normal watering), mild salt stress (S1, 200 mmol/L NaCl), moderate salt stress (S2, 400 mmol/L NaCl), and severe salt stress (S3, 600 mmol/L NaCl). *P. grandiflorus* seedlings grew to approximately 15 cm in height, after which NaCl or water was added as per the treatment. To ensure that salt solution was completely spread in dry soil, water control treatment was performed 5 days before the increase in salt solution. To avoid the effect of salt stimulation, 200 mmol/L NaCl was added daily until the predetermined level of each stress was reached. During the experiment, the temperature and humidity remained unchanged. Seedlings were collected at 8:00 am on the 30th day after treatment. The harvested *P. grandiflorus* seedlings were stored in a freezer and sent to the laboratory within 5 min. Before transcriptome sequencing and metabolite analysis, some *P. grandiflorus* samples were quickly cleaned, frozen in liquid nitrogen, and stored at − 80 °C. The remaining samples are used to measure biomass and physiological indicators.

### Determination of plant biomass and physiological index

For this experiment, 6 replicates (6 plants) of each treatment were washed with tap water to remove soil on the seedlings. After wiping the water off the seedlings with absorbent paper. The seedlings were heated in oven at 105 °C, after which the temperature was allowed to naturally drop to 65 °C and the seedlings were incubated at 65 °C for 48 h. Finally, we determined the constant dry weight of the above- and underground parts.

The content of MDA in the samples was determined using thiobarbituric acid (TBA) method to estimate the degree of lipid peroxidation [37]. Briefly, 0.1 g of the root was homogenized in 600 mL of 0.1% (w/v) trichloroacetic acid (TCA) solution and centrifuged for 15 min at 4℃ at 14,000 rpm. The supernatant was collected in a tube, and 0.5–1.5 mL of 0.5% (w/v) TBA in 20% TCA was added. The tube was boiled for 25 min and further immersed in ice water bath to complete the reaction. The absorbance of the supernatant was measured at 532 nm; the nonspecific absorption value at 600 nm was subtracted to calculate the MDA content. EL was determined as described previously [38]. Briefly, a fresh sample of *P. grandiflorus* was taken; the root was thoroughly washed with tap water, dipped into a glass tube containing 15 mL of deionized water with known electrical conductivity (EC), and vigorously rotated. After 24 h in dark, EC was measured using a Conductometer (HerisauMetrohm, Switzerland) before and after boiling for 20 min at 110 °C. The damage index was estimated using the following formula:

$${\rm{EL}}\,\left( {\rm{\% }} \right)\,{\rm{ = }}\,\left[ {\left( {{\rm{preboiling EC/postboiling EC}}} \right)\,{\rm{ \times }}\,{\rm{100}}} \right]{\rm{/initial}}\,{\rm{root}}\,{\rm{weight}}$$

Three replicates per treatment were measured.

Antioxidant enzymes: The activity of SOD was evaluated by measuring its ability to inhibit photochemical reduction of nitroblue tetrazole (NBT). The reaction mixture contained 100 μL enzyme extract, 50 mM potassium phosphate buffer (pH 7.8), 13 mM methionine, 75 μM NBT, 2 μM riboflavin, and 0.1 mM EDTA. In the 5000 lx light radiation of the reaction mixture for 15 min. One unit of SOD activity was defined as the amount of enzyme that caused 50% inhibition of the reduction of NBT as monitored by measuring absorbance at 560 nm and expressed as the unit of enzyme activity (U mg^− 1^ protein). POD activity was determined using guaiacol method. 28 μL of guaiacol was dissolved by magnetic heating agitator. After the solution was cooled, 19 μL of 30% hydrogen peroxide was added. It was mixed with 50 mL of 100 mmol/L phosphoric acid buffer (pH 6.0) to prepare the reaction mixture. Further, 0.5 mL of enzyme extract was added to 3 mL of reaction mixture to initiate the reaction. For this experiment, 0.01 change of A_470_ per min was considered as a POD activity unit, and POD was expressed as U mg^− 1^ protein. CAT activity was determined by measuring the depletion of H_2_O_2_ over 3 min at 240 nm [39]. The reaction mixture was prepared by mixing 0.2 mL enzyme extract, 1.5 mL phosphate buffer (pH 7.8), and 1 mL distilled water. CAT activity was expressed as U mg^− 1^ protein. As previously described [40], the activity of APX was measured based on the reduction in A_290_ of 1 mL of the reaction mixture within 1 min. The reaction mixture contained 50 mM monopotassium phosphate buffer (pH 7.0), 0.5 mM ascorbic acid, 0.1 mM EDTA, 0.1 mM H_2_O_2_, and 0.5 μL enzyme extract. APX activity was expressed in U mg^− 1^ protein.

Osmotic substances: The content of proline in *P. grandiflorus* was determined as described by Bates et al. [41]. In brief, the roots of *P. grandiflorus* (0.5 g) were placed into test tubes, and 5 mL 3% sulfosalicylic acid solution was added to each tube. The tube was placed in a boiling water bath for 10 min and filtered. The filtrate contained the extracted proline. To 2 mL of proline extract, 2 mL glacial acetic acid and 2 mL acidic ninhydrin reagent were added. The mixture was heated for 30 min in boiling water bath. After cooling, 4 mL toluene was added, shaken for 30 s, and allowed to stand for a while. The top layer was taken in a 10-mL centrifuge tube and centrifugated at 3000 rpm for 5 min. Total soluble sugar (TSS) was measured according to the method described by Irigoyen et al. [42]. In a mortar, 0.5 g of *P. grandiflorus* root was ground in 4 mL of 80% alcohol to form a homogenate. It was poured into a centrifuge tube, placed in a water bath at 80℃, continuously stirred for 30 min, and centrifuged for 10 min at 5000 rpm. The supernatant was collected. To it, 0.5 g active carbon was added and decolorized in 80℃ water bath for 30 min. After filtering, the filtrate contained sugar extract. To 1 mL of this sugar extract, 5 mL of anthrone reagent was added, mixed, boiled in a boiling water bath for 10 min, and cooled. The absorbance was measured at 625 nm.

### Transcriptome sequencing and data analysis

To study the changes in the transcription level of *P. grandiflorus* under salt stress, total RNA was extracted from the roots using standard extraction methods, followed by strict quality control of RNA samples. RNA integrity was accurately detected using Agilent 2100 bioanalyzer. The starting RNA of library was total RNA with total RNA ≥ 1 μg. PCR products were purified, and the cDNA library was finally obtained. After the construction of the library, Qubit2.0 Fluorometer was used for the preliminary quantification. The PCR products were diluted to 1.5 μg/μL, and the insert size was detected using Agilent 2100 bioanalyzer. After the insert size met the expectation, qRT-PCR was performed to accurately quantify the effective concentration of the cDNA library (the effective concentration of the library was > 2 nM) to ensure the quality. To ensure the quality and reliability of the data, the original data was screened to finally determine the content of Q20, Q30, and GC of the clean data. All subsequent analyses were high-quality analyses based on clean data. Finally, Trinity was used to splice the clean reads.

Gene functions were annotated according to the following databases: NR, NT, KOG/COG, KO, and Gene Ontology (GO). DESeq R package (1.10.1) was used for differential expression analysis of two conditions or groups. The genes with P < 0.05 and VIP > 1 found by DESeq were designated as DEGs. A q value < 0.005 and |log2| > 1 were set as the threshold for significant differential expression. We used GOseq and KOBAS software to perform GO function enrichment analysis on the differential gene sets. KEGG was used to analyze information at the molecular level. Large-scale molecular datasets (http://www.genome.jp/KEGG/) were generated by genome sequencing and other high-throughput experimental techniques, which were tested using Kobas software. DEGs were statistically enriched in KEGG pathway. Enrichment analysis revealed enrichment of all differential gene sets and upregulated and downregulated DEGs of each differential comparison combination.

### Extraction and analysis of metabolites

Metabolite extraction and analysis was performed using Liquid Chromatograph Mass Spectrometer (LC–MS). The freeze-dried *P. grandiflorus* samples were crushed with a stirrer at 60 Hz for 2 min. Further, 50 mg powder of each sample was transferred to a 2 mL EP tube and extracted with 1000 μL of methanol/water mixture (v:v = 3:1). The samples were vortexed for 30 s, sonicated on an ice bath for 15 min, and shaken overnight at 4 °C. All samples were centrifuged at 12,000 rpm for 15 min at 4 °C. The resulting supernatant was passed through a 0.22-μm filter membrane, diluted 10-fold in a methanol: water mixture (v:v = 3:1), vortexed for 30 s, and stored at − 80 °C until UHPLC-MS/MS analysis. Quality control (QC) samples were prepared by mixing equal amounts of supernatants of all samples.

The ultra-performance liquid chromatography separation was performed on a Waters ACQUITY UPLC HSS T3 column (100 × 2.1 mm, 1.8 μm). Mobile phase A was 0.1% formic acid in water, and mobile phase B was acetonitrile. The elution gradient was adopted. The column temperature was set at 40 °C. The autosampler temperature was set at 4 °C,and the injection volume was 2 μL [43, 44].

The high-resolution mass spectrometry data were converted to mzXML format using ProteoWizard and processed using MAPS software (version1.0) [45]. The preprocessing results generated a data matrix with retention time [46], mass-to-charge ratio (m/z) values, and peak intensities. The in-house MS2 database was applied for metabolite identification. In addition, the MRM data were processed [47, 48].

### Statistical analysis

GraphPad Software Inc, San Diego, CA, was used for one-way ANOVA and Tukey’s multiple comparison test. R statistical language was used for most of the statistical analyses in this study. Log2 normalization was used before statistical analysis. The effect of treatments on individual metabolites, transcripts, or physiological indexes in the experiment was tested using a one-way ANOVA corrected for false discovery rate (FDR) [49]. Principal component analysis (PCA) was performed using the R package “pcaMethods.” Pearson’s correlation analysis was performed using the R function “or test” in the “Statistics” package.

### Electronic supplementary material

Below is the link to the electronic supplementary material.

Supplementary Material 1

Supplementary Material 2

## Data Availability

Transcriptome raw data in this study have been uploaded to the National Center for Biotechnology Information Sequence Read Archive (SRA) database (https://www.ncbi.nlm.nih.gov/sra; accession no. PRJNA932949).

## Abbreviations

APX: Aseorbateperoxidase
CAT: Catalase
EL: Electrolyte Leakage
MDA: Malondialdehyde
POD: Peroxidase
SOD: Superoxide dismutase
