# Supplementary material for: Investigation of the response of Platycodongrandiflorus (Jacq.) A. DC to salt stress using combined transcriptomics and metabolomics
Source: BMC Plant Biol. 2023 Nov 25;23:589. doi: 10.1186/s12870-023-04536-w (PMC10675982; doi:10.1186/s12870-023-04536-w)
Supplement: Supplementary file 2 — Supplementary Material 2 [file 12870_2023_4536_MOESM2_ESM.docx]

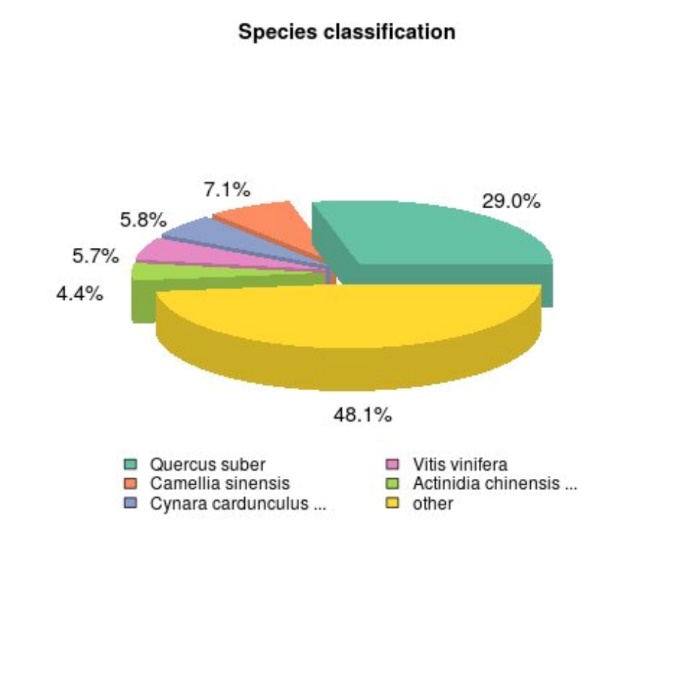


**Supplementary Figure 1** Species distribution of the top BLAST hits of *Platycodon grandiflorus* unigenes when the encoded proteins were queried against the NCBI nonredundant protein database.


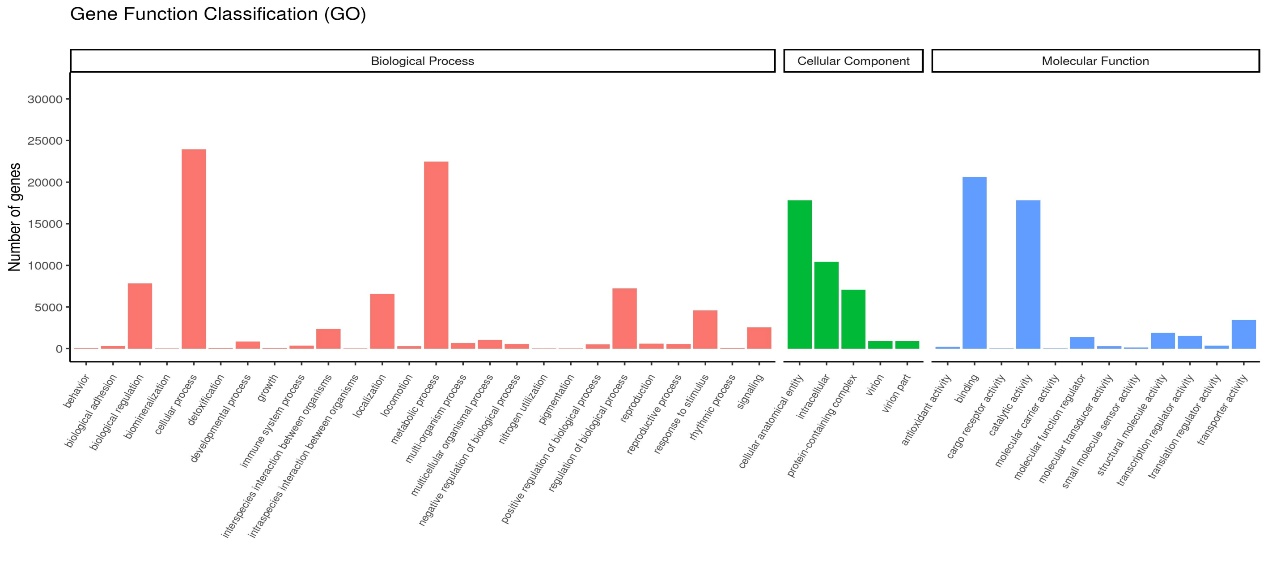


**Supplementary Figure 2** Gene ontology classification of the *Platycodon grandiflorus* unigenes.


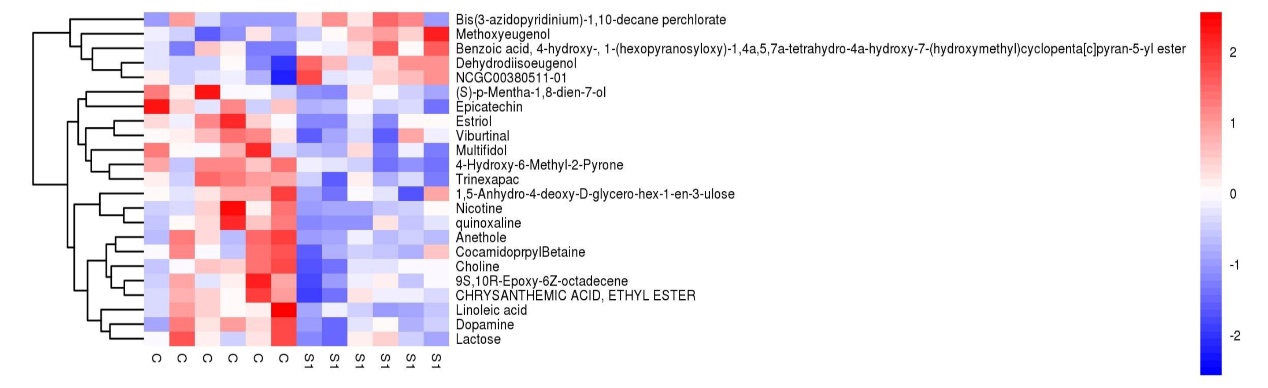


**Supplementary Figure 3** Hierarchical clustering heat map of differential metabolites under mild salt stress (S1). The abscissa in the figure represents different experimental groups, the ordinate represents the different metabolites compared in this group, the color blocks at different positions represent the relative expression amount of metabolites at corresponding positions, red represents the high expression of the substance content, and blue represents the low expression of the substance content.


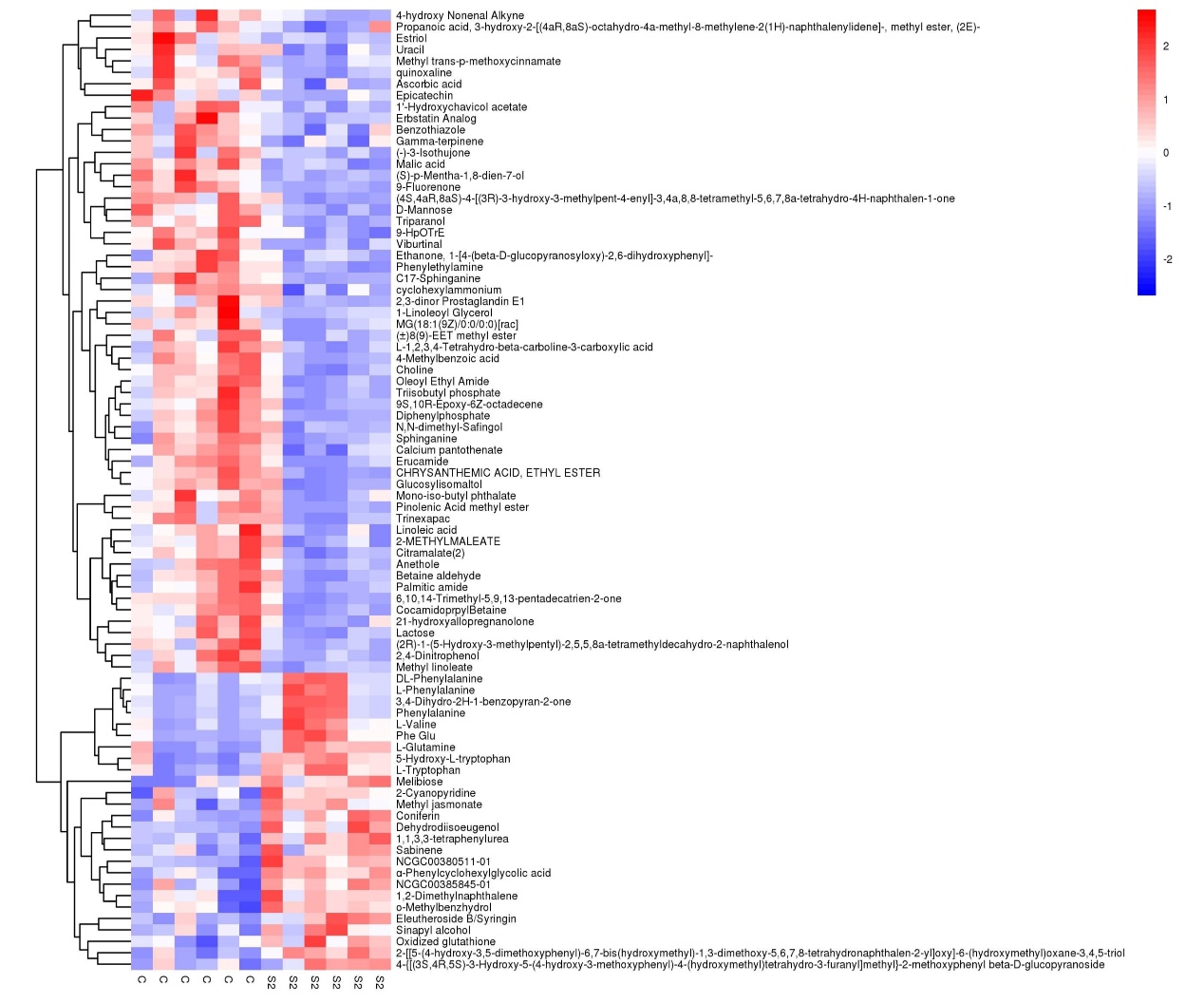


**Supplementary Figure 4** Hierarchical clustering heat map of (S2) differential metabolites under moderate salt stress. The abscissa in the figure represents different experimental groups, the ordinate represents the different metabolites compared in this group, the color blocks at different positions represent the relative expression amount of metabolites at corresponding positions, red represents the high expression of the substance content, and blue represents the low expression of the substance content.


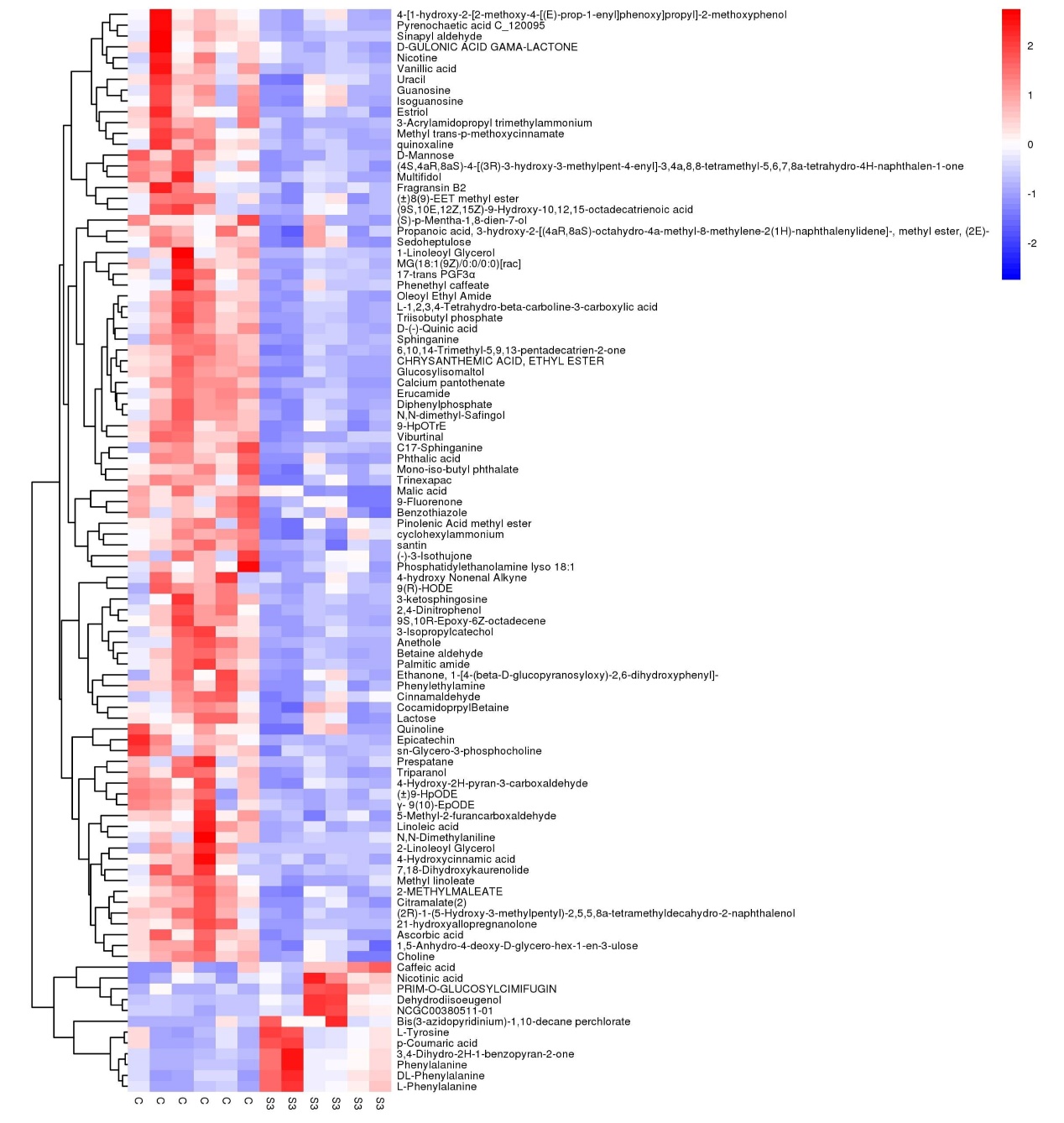


**Supplementary Figure 5** Hierarchical cluster thermogram of the differential metabolite (S3) under severe salt stress. The abscissa in the figure represents different experimental groups, the ordinate represents the different metabolites compared in this group, the color blocks at different positions represent the relative expression amount of metabolites at corresponding positions, red represents the high expression of the substance content, and blue represents the low expression of the substance content.


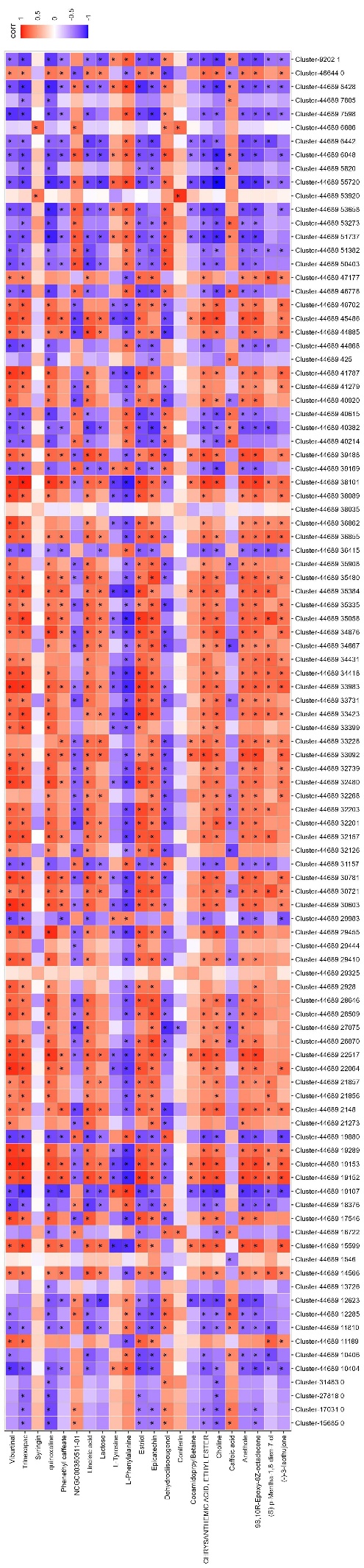


**Supplementary Figure 6** Correlation analysis of DEGs and differentially abundant metabolites under different salt stress treatments. A total of 101 DEGs and 22 differentially abundant metabolites were used for correlation matrix analysis. * p value < 0.05 of differentially abundant metabolites and genes.
